# Health system challenges and opportunities in organizing non-communicable diseases services delivery at primary healthcare level in Bangladesh: A qualitative study

**DOI:** 10.3389/fpubh.2022.1015245

**Published:** 2022-11-09

**Authors:** Ashraful Kabir, Md Nazmul Karim, Baki Billah

**Affiliations:** Department of Epidemiology and Preventive Medicine, School of Public Health and Preventive Medicine, Monash University, Melbourne, VIC, Australia

**Keywords:** health system challenge, health system dynamics framework, primary healthcare (PHC), non-communicable diseases (NCD), qualitative study

## Abstract

**Introduction:**

The weak health system is viewed as a major systematic obstacle to address the rising burden of non-communicable diseases (NCDs) in resource-poor settings. There is little information about the health system challenges and opportunities in organizing NCD services. This study examined the health system challenges and opportunities in organizing NCD services for four major NCDs (cervical cancer, diabetes mellitus, cardiovascular diseases, and chronic respiratory illnesses) at the primary healthcare (PHC) level in Bangladesh.

**Methods:**

Using a qualitative method, data were collected from May to October 2021 by conducting 15 in-depth interviews with local healthcare providers, 14 key informant interviews with facility-based providers and managers, and 16 focus group discussions with community members. Based on a health system dynamics framework, data were analyzed thematically. Information gathered through the methods and sources was triangulated to validate the data.

**Results:**

Organization of NCD services at the PHC level was influenced by a wide range of health system factors, including the lack of using standard treatment guidelines and protocols, under-regulated informal and profit-based private healthcare sectors, poor health information system and record-keeping, and poor coordination across healthcare providers and platforms. Furthermore, the lack of functional referral services; inadequate medicine, diagnostic facilities, and logistics supply; and a large number of untrained human resources emerged as key weaknesses that affected the organization of NCD services. The availability of NCD-related policy documents, the vast network of healthcare infrastructure and frontline staff, and increased demand for NCD services were identified as the major opportunities.

**Conclusion:**

Despite the substantial potential, the health system challenge impeded the organization of NCD services delivery at the PHC level. This weakness needs be to addressed to organize quality NCD services to better respond to the rising burden of NCDs at the PHC level.

## Introduction

Despite achieving remarkable advances in health outcomes in recent decades, non-communicable diseases (NCDs) remain a major public health challenge in Bangladesh (1). The prevalence of NCDs has increased substantially across the population of different ages (e.g., relatively young people), occupational groups, and those living in both urban and rural settings (e.g., rural and urban slums) (2–5). The Global Burden of Diseases study reported that the proportion of deaths from NCDs gradually increased from 43.4% in 2000 to 66.9% in 2015 (6). According to the available information, NCDs accounted for 67% of total deaths and shared approximately 64% of the disease burden in 2016 (7). As such the national STEPS (STEP wise Surveillance) survey reported a high prevalence of the behavioral, physical, and biochemical risk factors such as the use of tobacco products (43.7%), diabetes mellitus (12.7%), high blood pressure (13.2%), low physical activity, obesity, insufficient fruit and vegetable intake, extra salt intake. Overall, 70.9% of the adult population aged 18–69 years had at least one NCD-related risk factor (8). Furthermore, individual studies reported that NCD-related risk factors such as living a sedentary lifestyle, consumption of unhealthy diets, use of tobacco products (3, 9), and ongoing socio-demographic transitions may have fueled the shift in disease epidemiology. Due to these factors, the prevalence of NCDs will have a rising trend in the coming years unless appropriate strategies and programmatic measures are taken (10, 11). Recognizing the current NCD burden and future predictions, the government of Bangladesh has implemented various policies and strategies to address the rising burden of NCDs. The key aim of these controlling strategies is to mobilize the NCD services at the primary healthcare (PHC) level. The PHC system is the largest health service delivery system, and serves as the first point of contact for around 70% of the population (12).

The World Health Organization (WHO) defined the health system as “all organizations, people, and actions whose primary intent is to promote, restore or maintain health” (13), which are crucial for successfully adopting policies and implementing practical actions to improve health outcomes (14). Due to the weak health system, the organization of NCD services of NCD delivery services has remained a major challenge in low- and middle-income countries (LMICs) that hinders the designing and implementation of health programs/interventions (15–17). Limited capacity in various health system component(s) at the PHC level was reported as a barrier to the organization of NCD services delivery at the population level in LIMCs (18–20). Despite the growing emphasis on the need for health system research, a few studies have investigated whether and how health system factors affect the organization of NCD-related services delivery at the PHC level in LIMCs (21, 22). Similarly, in other LMICs, such as Bangladesh, the health system factors for the organization of effective NCD services delivery at the PHC level remain under-researched. The number of studies that investigated the health system aspects at the PHC level in the organization of NCD services delivery is very limited, and they focused on the hospital-based delivery of NCD services (23, 24). An earlier study reported various health system constraints in the implementation of NCD services in the NCD corners (dedicated NCD services) at the Upazila Health Complex (UHC) (the first level public hospital) (24), and a recently published study reported the implementation status of the NCD control program at the PHC level (23). However, these studies were based on the first-level hospitals in the country and/or included responses of the healthcare providers only. These studies lack insights from the other frontline healthcare providers, informal providers, and community members who are the major stakeholders of the healthcare system in Bangladesh. Therefore, the need for a comprehensive and deeper investigation into health system factors engaging a wide range of providers, stakeholders, and parties remains a high priority to better understand the health system challenges and opportunities in organizing NCD services at the PHC level.

Thus, this study aims to examine the health system challenges and opportunities in organizing effective NCD services delivery at the PHC level. The findings may be useful to support public health decisions in addressing the rising burden of NCDs in Bangladesh and similar settings elsewhere.

## Materials and methods

### Study design

The study adopts an exploratory qualitative design.

### Study time and settings

Interviews were conducted between May and October 2021. This study was conducted in four administrative districts in Bangladesh: Cumilla, Jhenaidah, Rajshahi, and Sylhet. These selected areas belong to the rural setting of the country. Although Bangladesh's health system is considerably uniform in terms of health service delivery, organization of the healthcare workforce, and logistics and supplies; varying socio-demographic characteristics, geographical elements, livelihood patterns, sociocultural norm, and practices may affect health outcomes (12). Considering these variabilities data were purposefully collected from participants with a wide range of roles, backgrounds, organizations, and geographical locations. It is worth mentioning that Bangladesh's health system is pluralistic–multiple actors and providers play roles in applying a mixed system of medical practices under the Ministry of Health and Family Welfare (25). A wide range of facilities and services are provided from primary to tertiary levels. According to the administrative structure, Bangladesh has approximately 87,310 villages, 40,977 wards, 4,553 Union, 490 sub-districts, 64 districts, 4 metropolitan cities, and 8 divisions (26). Primary healthcare services are concentrated at the sub-district (locally known as Upazila) level, largely provided through various Upazila-based healthcare facilities and frontline staff. Approximately 70% of the total population of the country residing in rural districts receive healthcare from the primary healthcare system as the first-line contact for health needs (10, 27). However, the primary healthcare services in the metropolitans and municipalities are largely organized through project-based targeted interventions and partnership programs between local governments (i.e., municipalities, and city corporations), NGOs, and donor agencies (s) under the Ministry of Local Government, Rural Development & Cooperation (28, 29). Due to various factors such as complex healthcare delivery mechanisms, target population (i.e., highly focused on the slums dwellers, poor neighborhoods), greater reliance on the private healthcare sector, resource mobilization and allocation modality, relatively small coverage, and study budget and field constraints, we decided to exclude metropolitan cities in this study as detailed in our protocol paper (12).

### Study participants and sampling strategy

We conducted 16 focus group discussions (FGDs) with people in the community members with one of the four major NCDs: cervical cancer, chronic respiratory illnesses (CRIs), cardiovascular diseases (CVDs), and diabetes mellitus; 14 key informant interviews (KIIs) with medical doctors, facility managers, and community leaders; and 15 in-depth interviews (IDIs) with local-level formal and informal healthcare providers such as kabiraj, faith healers, unqualified allopathic providers who are the major healthcare providers in the context of Bangladesh (Table 1) (30). Community leaders and faith leaders on some occasions serve as a member of the Community Group which is the management body of the Community Clinic (31). They might have possessed rich information about the NCD service delivery mechanism and the existing healthcare system due to their roles and social positions. We interviewed them to capture rich insights into the context, NCD-related service availability, service users' preference, and practice, access and coverage, and healthcare utilization which are important health system components. The FGDs helped to explore and understand the views, attitudes, and opinions regarding the existing NCD-related services and care. The IDIs helped to explore the frontline healthcare providers' opinions and experience regarding the organization of NCD services delivery and care. The KIIs provided additional insights into policy formulation and implementation, as well as management-related information and views regarding NCD-related services and care. We employed purposive sampling to select the participants, which is a commonly used sampling strategy in qualitative research and has been used previously in health research in the context of Bangladesh (32, 33). In the initial step, we approached potential participants and explained the study's objectives and purpose. We applied the following inclusion criteria in selecting the potential interviewees: people aged 18 years and above; time and availability; and voluntary participation. Based on the literature and extensive experience of the research team in similar contexts and issues, we planned to conduct 16 FGDs, 16 IDIs, and 16 KIIs to reach data saturation, as detailed in our study protocol (12). However, we reached the point of data saturation by conducting 16 FGDs, 14 KIIs, and 15 IDIs, as no new information, dimensions, or concepts emerged, according to the proposition by Guest et al. (34). We followed a stepwise procedure to reach the point of data saturation. Multiple interviewers simultaneously conducted interviews and then developed initial/axial coding. After conducting two-thirds of the interviews, the research team independently developed the codes and identified that no new information, dimensions, or themes emerged. When the research team realized that no new themes were being generated, they agreed that data saturation had been achieved. In this process, we followed a few basic norms in selecting the participants with the principles of ensuring there was maximum variation of organizations, age, sex, occupations, and administrative roles; an iterative process (moving back and forth between data collection and coding); and reflexivity (assessed self-roles of the interviewers). We used semi-structured interview guidelines. Before commencing the main study, a pilot study was undertaken outside the original sites. The FGD guidelines were piloted in Bagha Upazila (sub-district) of Rajshahi, while the KII and IDI guidelines were tested in Kaliganj Upazila of Jhenaidah district. The pilot tests included participants with similar backgrounds and roles (i.e., front-line healthcare providers, health managers, and community members). This pilot study aimed to determine the acceptability and practicability of the questions/topics, comfortability of conversion, and estimate time required for interviewing each category of participant. Necessary changes were made in the interview guides based on the findings of the pilot tests.

**Table 1:** Data collection methods and study participants.

**Methods**	**Participant(s)**			
	**Cumilla**	**Jhenaidah**	**Rajshahi**	**Sylhet**
IDIs (*n* = 15)	IDI1: with health assistant (*n =* 1); IDI2: with family welfare visitor (*n =* 1); IDI3: with community healthcare provider (*n =* 1); IDI4: with kabiraj (*n =* 1)	IDI1: with family welfare visitor (*n =* 1); IDI2: with village doctor (*n =* 1); IDI3: with sub assistant community medical officer (*n =* 1); IDI4: with faith healer (*n =* 1)	IDI1: with Sub assistant community medical officer (*n =* 1); IDI2: with community healthcare provider (*n =* 1); IDI3: with health assistant (*n =* 1)	IDI1: with community healthcare provider (*n =* 1); IDI2: with faith healer (*n =* 1); IDI3: with Sub assistant community medical officer (*n =* 1); IDI4: with faith healer (*n =* 1)
KIIs (*n =* 14)	KII1: with independent consultant (*n =* 1); KII2: with civil surgeon (*n =* 1); KII3: with community leader (*n =* 1)	KII1: with upazila health and family planning officer (*n =* 1); KII2: with medical officer (*n =* 1); KII3: with community leader (*n =* 1); KII4: with medical officer (*n =* 1)	KII1: with independent consultant (*n =* 1); KII2: with residential medical officer (*n =* 1); KII3: with medical officer (*n =* 1); KII4: with Civil Surgeon (*n =* 1)	KII1: with medical officer (*n =* 1); KII2: with upazila health and family planning officer (*n =* 1); KII3: with residential medical officer
FGDs (*n =* 16)	FGD1: with community members (in each district, *n =* 4)			

### Data collection procedure

Semi-structured interview guides were used for collecting data (Supplementary material 1). Interview schedules were developed to conduct interviews on time. Based on the participants' availability, the interviewers visited the data collection sites. The interviewers created a good rapport by introducing themselves, expressing gratitude, explaining the research objectives and purpose, and encouraging the participants to ask questions or express their thoughts. The interviews were conducted in Bangla, a language spoken by both the interviewers and participants. Formal written consent was obtained. In this process, an explanatory statement was provided to the participants and allowed them to read and ask questions. This document contained information about the background and purposes of the study, risks and benefits, roles and expectations, the freedom to participate, contact information for further queries, confidentiality, and anonymity. Upon their agreement to participate, participants are required to read and sign a consent form. The interviews were audio-recorded after obtaining the consent of the interviewees. Two interviewers who graduated in social science and medicine conducted the interviews, and two research assistants (social science graduates) took the field notes. The interviewers were trained in qualitative research methods and had extensive experience in applying various data collection tools and techniques in similar settings. It took ~45–60 min to conduct each FGD, and 30–45 min to conduct each IDI and KII.

### Data analysis and theoretical framework

**Figure 1:**
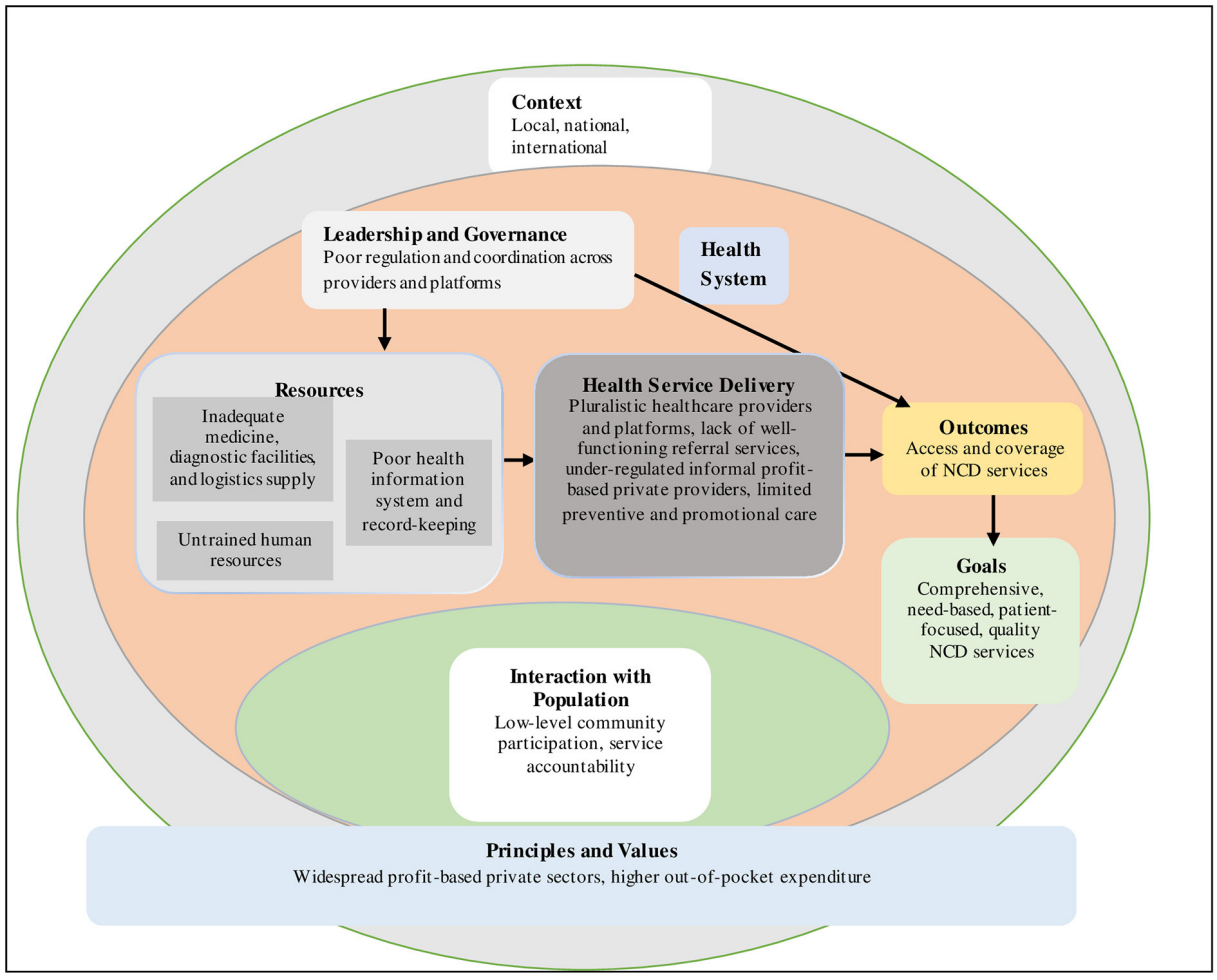
Health system dynamics framework on health system challenges to organize NCD services at the PHC level.

The health system dynamics framework was used as an analytical and conceptual tool to explore and understand a range of health system factors that influence the organization of NCD services delivery at the PHC level. It is worth mentioning that the WHO's health system framework has been predominantly applied in health system research, which describes “six” key elements or “building blocks” to describe, analyze, and plan for healthcare service delivery among the population (35). However, the WHO's framework is viewed as having limited capacity to comprehensively explain how and whether different health system elements within a broader societal context are interacted with and influenced, how population/individuals' behavior and choices, and the process impact this mechanism (36). To provide a more comprehensive understanding of system interactions, the “health system dynamics framework” was proposed by Van Olmen et al. (37) which consists of ten (i.e., goals and outcomes, values and principles, service delivery, population, context, leadership and governance, finance, human resources, infrastructure and supplies, knowledge and information) elements under broader societal context. This framework has been considered the best suited for the current study as it incorporated “building blocks” health system elements from the WHO's building block health system framework and concurrent literature. The health system framework was used as an initial coding guide. The newly emerging codes were subsequently added to the framework to build our model of factors influencing the organization of NCD services at the PHC level (Figure 1).

Guided by the health system dynamics framework and research objectives, we applied a hybrid approach combining deductive and inductive coding procedures (38) to identify the themes related to the organization of NCD services delivery at the PHC level. The “consolidated criteria for reporting qualitative studies: 32-item checklist” was followed to report the findings (39). We followed a stepwise procedure to perform the thematic analysis (a) we developed a list of priori codes based on the information available in the literature and research team members' understanding and experience that have been gained through their engagement in their respective study domains, (b) getting familiarized ourselves with the textual data through repeated reading, (c) beginning priori and posterior codes and adding them to the code list based on the information from the transcripts, (d) reviewing and sorting all coded data and formatting a thematic matrix, (e) collapsing the priori and posterior codes under the final themes and sub-theme, and (f) reporting findings under the themes. This thematic analytical procedure enabled us to frame uncategorized themes which were effective to explore associated factors that influenced the delivery of NCD services at the primary healthcare level. Nevertheless, organizing and merging the codes/themes that appropriately capture the participants' views and comments were the major challenge in this approach. Formal discussions among the independent coders were carried out to resolve any disagreements so that a consensus was reached.

## Results

### Characteristics of the participants

This section presents the socio-demographic profiles of the participants and is followed by the thematic analysis. Table 1 shows the various data collection methods, including 15 IDIs, 14 KIIs, and 16 FGDs.

The detailed background characteristics of the participants are shown in Table 2. The combined mean age for FGD participants was 51 years (±8). About one-third (33/96) of the participants did not have formal education, and only seven participants had more than year ten level education. Majority of the participants across the sites were married (61/96), and the majority of the participants lived in a joint family (69/96). Informal occupations (e.g., agricultural laborers, homemakers, students, businessmen, farmers, etc.) were predominant across sites (79/96). Almost two-thirds (74/96) of the participants were Muslim. The highest number of participants (52/96) had a monthly family income of >10,000 BDT (90 BDT is almost equal to 1 dollar during the interview time). More than half (52/96) of the participants had multiple NCDs (e.g., CRI, cancer, CVD, and diabetes), and the majority of the participants had NCDs for over 5 years. The mean age of the IDI participants was 37 years (±5); eight of them were female. All the IDI participants were local-level healthcare providers. Six of them had more than10 years of professional experience. The mean age of the KII participants was 42 years (±9); nine were male. The majority of KII participants were healthcare providers (8/14), and the remainder were involved in policy planning (2/14) or healthcare activism (2/14).

**Table 2:** Background information of participants in FGDs, IDIs, and KIIs.

**Focus Group discussions (*n =* 16, participants: 92)**					
	**Study sites**				**Combined**
**Characteristics**	**Cumilla**	**Jhenaidah**	**Rajshahi**	**Sylhet**	
**Age in years (mean** **±SD)**	55 (±12)	54 (±10)	49 (±5)	43 (±11)	51 (±8)
**Gender (** ***n*** **)**					
Male (*n*)	9	10	12	10	41
Female (*n*)	11	15	14	11	51
**Education (** ***n*** **)**					
No formal education (*n*)	10	9	7	7	33
Primary school (grades I–V) (*n*)	4	8	9	11	32
Secondary school (grades VI–X) (*n*)	4	8	5	3	20
Higher (>grade X) (*n*)	2	-	5	-	7
**Marital status (** ***n*** **)**					
Married (*n*)	16	16	14	15	61
Unmarried (*n*)	3	4	5	2	14
Widowed (*n*)	1	5	7	4	17
**Family type (** ***n*** **)**					
Nuclear (*n*)	5	10	2	6	23
Joint (*n*)	15	15	24	15	69
**Occupation (** ***n*** **)**					
Formal (*n*)	4	3	2	4	13
Informal (*n*)	16	22	24	17	79
**Religious identity (** ***n*** **)**					
Muslim (*n*)	16	25	17	16	74
Hindu (*n*)	4	-	6	4	14
Others (*n*)	-	-	3	1	4
**NCD status (** ***n*** **)**					
Developed at least 1 NCD (*n*)	6	14	11	9	40
Multiple NCDs (*n*)	14	11	15	12	52
**Duration of suffering from NCDs (n)**					
< 3 years (*n*)	3	3	2	2	10
3–5 years (*n*)	9	3	3	7	22
>5 years (*n*)	8	19	21	12	60
**Characteristics**	**In-depth interviews (*****n** =* **15)**			**Key informant interviews (*****n** =* **14)**	
**Age in years (mean** **±SD)**	37 (±5)			42 (±9)	
**Gender (** ***n*** **)**					
Male (*n*)	7			9	
Female (*n*)	8			5	
**Type of provider (** ***n*** **)**					
Healthcare professional (*n*)	-			8	
Healthcare manager (*n*)	-			2	
Policy planner/independent consultant (*n*)	-			2	
Civil society/Non-Governmental Organization (NGO) worker (*n*)	-			2	
**Occupation (** ***n*** **)**					
Health assistant (*n*)	2			-	
Family welfare visitor (*n*)	2			-	
Community healthcare provider (*n*)	3			-	
SACMO (*n*)	3			-	
Kabiraj/faith healer (*n*)	3			-	
Village doctor (*n*)	1			-	
**Length of service/experience (** ***n*** **)**					
< 5 years (*n*)	5			3	
6–10 years (*n*)	4			4	
>10 years (*n*)	6			7	

### Thematic analysis

The dominant recurring themes reflecting the health system dynamics framework were identified and reported (Figure 1). The thematic analysis revealed a range of health system factors that challenged the organization of NCD service delivery at the PHC level. The major themes were as follows: health service delivery; infrastructure and supplies; human resources; knowledge and information; leadership and governance; and values and principles.

### Health service delivery

The availability of NCD services has increased at the PHC level. Diverse healthcare delivery platforms and providers offered NCD services within the PHC system, including governmental, private-for-profit, NGO, and private healthcare practitioners consisting of a wide range of informal providers. One of the participants stated:

“*NCD-related services are more available around us. Public facilities, private clinic, NGO clinic, village doctor offices, and individual providers (e.g., kabiraj, hakim, faith healer) can be reached in the locality, which was not possible in the past.”* (An FGD participant from Jhenaidah)

Healthcare providers at different platforms practiced various methods of treatment. Modern medicine known as allopathy is the dominant method in government healthcare facilities, private facilities, and a large number of informal providers such as village doctors' chambers, whereas informal providers practiced the traditional method using herbal plants, creepers, or anything similar that was available in the community. In a few cases, providers in government healthcare facilities had received NCD-related training and tried to follow relevant treatment guidelines and protocols during service provision. However, the guidelines for diabetes mellitus and hypertension were widely followed in facilities at the PHC level as nearly a quarter of KIIs and IDIs participants mentioned. Guidelines for the management and treatment of CVDs, asthma, and chronic obstructive pulmonary disease were rarely followed. Although the guidelines for cervical cancer screening and management were introduced in the PHC facilities, it was found that a higher number of providers were not adhere to them due to the lack of orientation and higher patient load. One of the participants stated:

“*Healthcare providers at the PHC facilities have very limited scope to follow the relevant guidelines and protocol in managing and treating NCDs due to the high patient load and lack of proper orientation. Among them, guidelines for diabetes and hypertension are more likely to be followed. Guidelines for asthma, cardiovascular diseases, and cervical cancer screening are less likely to be followed.”* (A KII participant from Jhenaidah)

However, informal providers received only a limited amount of NCD-related training and rarely followed guidelines in managing NCDs due to the lack of knowledge and training regarding the related guidelines. One participant stated:

“*I am not sure if there are guidelines and protocols for diabetes and/or hypertension. Say if a diabetes patient comes or a heart patient comes, then... you will eat these foods, you will walk 30 minutes in the morning, etc. We already tell patients about these in the ward when we counsel them. But if there are proper guidelines and protocols, it will be good.”* (A village doctor from Rajshahi)

Similarly, a higher number of providers in private and NGO facilities were found to be non-compliant with NCD-related guidelines and protocols due to the lack of orientation and time constraints. Providers at government healthcare facilities have a huge patient load, and often a shortage of human resources limits the chance to comply fully with relevant guidelines and protocols. One participant stated:

“*The healthcare providers at the government facilities are aware of the NCD-related treatment guidelines, such as guidelines for diabetes and hypertension. However, it is very hard to follow these on many occasions due to high patient loads, time limitation, and shortage of manpower. We provide services to more than three times the number of patients than the capacity, and it's hard to pay detailed attention to the guidelines.”* (A KII participant from Rajshahi)

However, almost half of the participants in the KIIs and IDIs stated that healthcare providers are much more aware of related guidelines and usually followed them when managing NCDs at the public healthcare facilities. One participant stated:

“*We follow the protocol for managing hypertension and/or diabetes. For example, when the patient comes for the first time, we consider the patient's age, gender, family history, lifestyle-related information, etc., and take their blood pressure. Based on this information, we decide on appropriate treatments or recommendations.”* (An IDI participant from Jhenaidah)

Weak or no referral services were identified as a critical barrier to managing NCDs in the PHC system. The KII and IDI participants reported that despite hypertension and diabetes protocol emphasizing screening at community clinics (CCs) and referral and back-referral to the NCD corner of the UHC for confirmation and better management of NCDs, a functional referral system has not yet been established at the PHC level. The lack of a functional referral and back-referral system resulted in the irrational patient load on the higher-level healthcare facilities and quality NCD care. One participant reflected:

“*If a functional referral system existed, there would be better NCD management in the PHC system. We need to establish a functional referral system, as it has been emphasized in the relevant policy document in practice to better provide NCD services.”* (A KII participant from Jhenaidah)

Similarly, no participant from the FGDs reported that patients have been referred to higher-level healthcare facilities. All the FGD participants stated that it depends on whether the patient would like to seek care from the higher-level facilities/specialist. However, the local-level providers such as CCs and union health centers had never arranged a referral. According to one participant,

“*If any patients feel that the services of the local-level providers at the CC or union health center were not effective, they seek care from other providers and make their own choice and arrangement. No referral is made.”* (An FGD participant from Sylhet)

### Infrastructure and supplies

Supplies and logistics were identified as key barriers to effective NCD management at the PHC level in sites and methods. Although the supplies and logistics support have been increased substantially at the PHCs in recent years, specifically by establishing NCD corners at the UHCs, there is a critical shortage of medicines, diagnostic facilities, and trained human resources. The shortage of supplies resulted in the poor quality of care and created mistrust of facilities among the patients with NCDs. One participant stated:

“*The list of essential medicines for NCDs is good, but there is a huge gap between the demand and supply.... We have a supply of five packets (50 tablets) of Salbutamol for treating asthma. If I give ten tablets to a single patient, I can at best give this medicine to five patients.... Monthly, we have 15–20 patients. Therefore, we experience a huge shortage of medicine.”* (An IDI participant from Rajshahi)

Similarly, the community members reported that inadequate supplies of medicines were a key barrier that often resulted in non-adherence to medication or practicing self-management at home using herbal plants, creepers, or anything similar that was available in the community. Non-adherence to medication was more common in patients with NCD with the poor socioeconomic status worsened their medical condition and led to the development of complications/comorbidities, and increased disabilities and deaths. One participant stated:

“*The distribution of these medicines for whole months at CCs is finished in just 2–3 days of supply, and for the remaining days of the month patients have to pay out-of-pocket. Those who are poor cannot afford it from their own pockets and stop taking medicines.”* (An FGD participant from Jhenaidah)

Similarly, the limited diagnostic facilities were mentioned as a health system barrier at the PHC level. Despite the plan to provide basic equipment in the NCD corners, the supplies of this equipment had not yet arrived. Furthermore, full use of the available equipment was not possible due to the lack of trained and regular human resources. The patients were often asked to undergo basic diagnostic tests at private facilities, and this was not affordable according to the majority of the participants. Limited diagnostic facilities or their absence in the PHC-level facilities led to the patients seeking care from higher-level facilities (e.g., wealthy patients) or receiving incomplete treatment (e.g., poor patients). One participant stated:

“*In our NCD corner, there is no diabetes machine and no electrocardiogram system. If these are provided, it will be beneficial for the patients. Many patients cannot afford testing outside and are therefore unable to continue the treatment regularly; ultimately, the medical condition causes a complication.”* (A KII participant from Rajshahi)

Similar views were shared by an FGD participant:

“*They should keep at least basic instruments functional to treat NCDs, but they don't have them.... There is no test facility, and patients are asked to undergo the test at the private facilities, which many of us cannot afford.”* (An FGD participant from Sylhet)

### Human resources

Insufficient trained human resources were noted as a key barrier for all types of PHC facilities. More than two-thirds of the frontline healthcare staff, and almost all informal providers did not receive in-service training (formal training) on the detection, treatment, and management of major NCDs. Poor professional training on the definition and classification of hypertension (e.g., prehypertensive, hypertensive, various stages of hypertensive), diagnosis and assessment of diabetes and hypertension (e.g., medical history, physical examination, and simple laboratory investigation), treatment (antihypertensive medication), non-pharmacological management such as health promotion activities, health diets, tobacco cessation, reduction of salt intake), and referral services (referral, back referral, and follow-up) were commonly reported in all sites. Facilities commonly have a shortage of approved posts due to the prolonged vacancies or lack of proper training to manage NCDs. Vacancies in facilities-based providers (e.g., doctors, nurses, paramedics, technicians, health educators, SACMO) and community-based field workers (e.g., health assistants, family welfare assistants) resulted in poor delivery of NCD services and care. These human resources constraint affected the preventive service, which is an important component of effective NCD management. One participant stated:

“*We have two types of patients with NCDs: those with diabetes and those with hypertension. Currently, we have a shortage of medical officers. There is no doctor with NCD training here.”* (A KII participant from Rajshahi)

Another participant raised a concern about the challenges of providing preventive services for better NCD management due to the lack of trained and designated healthcare staff. He stated:

“*Major NCDs, for example, diabetes, hypertension, and cardiovascular diseases, are not medicine-oriented diseases. They require something more than medication, which is called lifestyle modification. Health education, counseling, and awareness building are more important to prevent and control NCDs. We are unable to provide this support due to a lack of trained and designated staff. Only emphasizing treatment can never be better management of NCDs.”* (A KII participant from Cumilla)

### Knowledge and information

The lack of using electronic medical record systems was reported as a common barrier to better NCD management at the PHC level. Both government and private healthcare facilities used paper-based medical information records that were provided by the respective healthcare facilities. These paper-based documents were usually provided when the patients sought care at their respective healthcare facilities. Some patients stated they usually hold these documents (one page of a small sheet of paper), where the doctor usually prescribes medicine and/or provides a diagnosis. It was reported that patients were usually unaware of the need to keep this record with care, and on many occasions, they failed to bring it with them during the follow-up visits. One participant stated:

“*Prescriptions (paper-based and hand-written) are rarely preserved at home. People usually do not take care when the medicine is taken.”* (A KII participant from Rajshahi)

This lack of facility-held medical records was also reported as the key limitation for making an informed clinical decision, tracking the follow-up, and achieving continuity of care. Although patients were expected to preserve and bring the one-page sheet of paper to the follow-up visit, a very small number of them comply with this expectation due to a lack of awareness. One participant stated:

“*The facility-held electronic information system had not yet been established. It may take time to establish an effective information system due to the lack of resources and manpower. We maintain a registry for patients with NCDs, as well as for those with other diseases.”* (A KII participant from Jhenaidah)

Another concern was that no population-level surveillance data was generated or used to assess NCD prevalence and risk factors or prepare a management plan within the PHC system.

### Leadership and governance

Poor steering capacity within the local-level stakeholders and parties was identified as a barrier to better NCD management. As mentioned earlier, there are multiple healthcare providers, including formal and informal sectors, within the PHC system, and therefore effective and systematic coordination is required to better plan for organizing NCD services. The informal healthcare providers, including village doctors, of which there is a large number and who are influential in the sector, and private providers (private/NGO facilities) are under-regulated. The majority of informal providers were not aware of the respective protocol/guideline for treating NCDs. The local-level healthcare managers lacked an effective mechanism for coherent vision and common planning for management of NCDs. One participant stated:

“*There is a large number of informal care providers, and they had a greater influence within the community for many years, but many of them are not fully aware of the existing protocol/guideline for treating NCDs. They need to be oriented properly and coordinated regularly for better NCD management at the local level.”* (A KII participant from Cumilla)

Furthermore, there was a lack of multi-sectoral collaboration and coordination among other stakeholders, which the NCD-related policy documents emphasized. The existing NCD-related documents emphasized the collaborative efforts between the stakeholders, parties, and communities known as the “whole of society approach” to influence public policy and increase awareness of NCD risk factors such as tobacco use, unhealthy diets, physical inactivity, and poor air quality. A local-level initiative involving the stakeholders and communities was reported as a common barrier in all methods and sites. According to one participant,

“*We have never heard of anything about NCDs, no campaigning, no participation, no discussion. We have never shared our views or thoughts on any platform.”* (A KII participant from Sylhet)

### Values and principles

The healthcare system is greatly shaped and guided by the values and principles within the context. The healthcare system in the country is pluralistic, and the private sector/practitioners are an influential component. The private healthcare facilities are operated on a for-profit principle that incurs higher out-of-pocket costs. The majority of participants shared a concern about the ever-increasing healthcare cost at the private facilities that constrains their access. Additionally, the increase in medicine costs, consultation fees, and diagnostic costs exerted pressure on many families, resulting in non-adherence to medicine or incomplete treatment. One participant stated:

“*Values and principles greatly influence the health-related policy that ultimately guides the organization and management of the healthcare of the population. The investment in public healthcare is inadequate, and the policy supports the profit-based health service provisions. Currently, over 70 percent of healthcare expenses are out-of-pocket, which limits the access to healthcare, especially for people with low income.”* (An IDI participant from Rajshahi)

### Perceived actions needed to improve the NCD services

The participants emphasized several measures to strengthen the NCD management actions and initiatives. The existing NCD responses are more focused on the diagnosis and treatment with little attention paid to the health promotion and preventive activities. Diagnosis and treatment-focused initiatives are helpful to those with chronic conditions, but preventive and health promotional activities are needed to avoid or delay the onset of this condition. It was perceived that there was a high need for a community-based population-level preventive initiative at the PHC level. One participant stated:

“*Our responses are more focused on the diagnosis and treatment of NCDs, but every year the prevalence increases. It is alarming that relatively young people with low socioeconomic status are developing NCDs.... NCDs are not diseases of wealthy people anymore. Promote and preventive interventions should be focused on more to tackle this epidemic.”* (An IDI participant from Jhenaidah)

Another participant stated:

“*Health promotion and prevention activities should be more extensive to make people aware and adopt a healthy lifestyle to fight NCDs.”* (A KII participant from Rajshahi)

The majority of participants noted that the facility-based NCD responses (mostly diagnosis and treatment) seriously lacked health education, counseling, and awareness-related interventions. Patients with NCD who attend the healthcare facilities had received little education on and had limited awareness of self-management, lifestyle modification, medication adherence, and the promotion of healthy behaviors at the healthcare facilities in the existing practice. One participant stated:

“*Healthcare providers do not have the scope to educate the patients at the facilities level due to a lack of manpower and time constraints. We need to mobilize more resources to extend this type of service.”* (A KII participant from Jhenaidah)

The capacity building of the large body of informal healthcare providers and meaningful coordination across diverse providers and platforms were emphasized to mitigate the existing resource constraints at the PHC level. It was emphasized that the existing guidelines and associated policy initiative need to be translated into practice to ensure quality and comprehensive NCD services. One participant stated:

“*The government of Bangladesh has formulated almost all of the policy documents to organize quality, and comprehensive NCD services at the PHC level. But what they lack is translating these into practice.”* (A KII participant from Rajshahi)

## Discussion

Using a health system dynamics framework, this study aimed to uncover how and which factors influence the organization and delivery of NCD services at the PHC level. Data from multiple sites and participants identified a wide range of factors situated on different levels of the health system dynamics framework. This study identified challenges and gaps in organizing and delivering NCD services related to five elements of the PHC system: health service delivery, infrastructure and supplies, human resources, knowledge and information, leadership and governance, and values and principles that guide the whole system.

Our findings revealed that NCD services have been increasingly mobilized at the PHC level through health system strengthening initiatives. These findings were similar to those of other recently published studies (24); however, these services are still considered to be suboptimal in terms of the increasing demand. Bangladesh's health system at the PHC level is inherently pluralistic, and the informal healthcare providers have been the dominant component. A previous study showed that providers constitute more than half of the healthcare workforce (e.g., 44% informal providers, 16% village doctors and drug salespeople) who possessed poor professional knowledge, which affects quality healthcare services (30). In line with the above-mentioned study, our findings suggest that the informal care providers, including the village doctors, drugstore salespeople, and faith healers, often received no training and are largely unaware of the existing treatment guidelines and protocols for major NCDs. The level of trust and acceptance of healthcare offered by informal healthcare providers are important predictors of health service utilization. There is a mix of findings regarding how the level of trust impacts the healthcare service provided by informal healthcare providers. A systematic review study reported that despite inadequate professional training, informal healthcare providers possess a high level of trustworthiness in the community due to perceived accountability, affordability geographic and social proximity to patients (40). In parallel, distrust often resulted in lowered preference and willingness to seek care from informal healthcare providers (41). Our study did not have much scope to explicitly explore how extent distrust affected the preference and willingness of seeking healthcare service from informal healthcare providers characterized by poor professional training and skills due to the objective of the study. A further study might be interesting to explore how and whether distrust affects the utilization of healthcare seeking from informal healthcare providers. The lack of proper orientation regarding the necessary treatment guidelines and protocols often resulted in suboptimal or poor NCD service delivery, as noted in previous studies (42, 43). Moreover, Bangladesh's significant health outcomes in recent decades have been attributed to the existence of a large number of informal healthcare providers that favored successful adaptation of numerous community-oriented targeted health programs/interventions through the pluralistic health system (44, 45). However, we argue that these improvements mostly occurred in the domains of maternal, infant, and child health; family planning and population control; immunization, and nutrition and vitamin supplementation that were prioritized in the major policy documents. Policy priorities duly facilitated numerous initiatives to engage informal and semi-qualified healthcare providers to be oriented and equipped with treatment/service delivery specifically by designing and implementing numerous vertical health programs in the above-mentioned domains (46). In parallel, NCDs were less prioritized in health-related policy planning, as the first sector-wide approach (SWA) in 1998–2003 did not prioritize NCDs with the health and population sector program, and the second SWA (2003–2011) did not have an NCDC operation plan (23). Although the third SWA (2011–2016) and fourth SWA (2017–2022) emphasized the need for mobilizing NCD services at the PHC level, there is a lack of proper planning for program designing and implementation and a monitoring system. Furthermore, due to the disease management approach, NCDs most often require extended, or even life-long healthcare support, early case findings/identification, psycho-social promotion, risk factor identification, regular medical support, adherence to medical procedures and treatment self-management, and behavior modifications (47). The current large-scale informal providers characterized by poor professional training and skills and a lack of proper orientation regarding the necessary treatment guidelines and protocols may not contribute positively to the quality NCD service delivery at the PHC level. We argue that informal healthcare providers need to be better trained, oriented and inclusive and better regulated in the management of NCDs.

The constant shortage of medicines, diagnostic facilities, equipment, and trained human resource remains a big challenge in NCD service delivery, which is a common feature of the PHC system in many LMICs, including Bangladesh (20, 48, 49). It was reported that PHC levels across the south Asian and sub-Saharan countries have insufficient supplies of medicines, basic amenities, diagnostic facilities, and workforce (50). In line with our review paper, the findings of this study revealed that insufficient resource mobilization is affecting NCD service delivery that needs to be emphasized to make the health system more responsive (48). Functional referral services and record-keeping are the integral components of a strong health system that ensures patients have access to the best possible higher-level facilities, utilize resources, and maintain the standardized process in service provision (51). However, the lack of a proper referral service and the weak medical record-keeping system are notable shortcomings that affected the rational and need-based utilization of healthcare services. The absence of a well-functioning referral system resulted in excessive use of secondary- or tertiary-level care facilities that allowed patients to directly use higher-level healthcare facilities to manage even minor and/or general ailments (52, 53).

In the past two decades, the growth of private healthcare sectors increased substantially and led to the expansion of services at the sub-districts level. Aligning with the profit-based values and principles, these profit-based private small and medium-sized healthcare facilities gradually increased and filled the shortage of NCD services in the public health sectors (54). Despite their crucial roles in providing healthcare services, compliance with various regulations remains a noticeable shortcoming for delivering NCD services (55), which is similar to the finding of a study conducted in India that the poorly regulated private healthcare sector appeared to be a systematic barrier to healthcare services in the primary healthcare system (16). Profit maximization became the central guide for operating healthcare delivery services and affected the access to and the quality of services. In many cases, the profit maximization views of the providers contributed to allocating inadequate time for diagnosis of diseases; charging high fees/charges; placing less emphasis on preventive and promotional health, health education, and counseling services; suggesting unnecessary diagnostics; and providing irrational practices that remain substantial challenges for NCD services (18, 55).

In line with a review study (20), our data revealed that the poor stewardship and coordination capacity of the local healthcare leaders (sub-district/district level government healthcare managers) leads to a lack in facilitating meaningful coordination across the stakeholders and parties specifically with private and informal providers in strengthening the PHC system as a whole system. The poor stewardship and coordination and the lack of proper monitoring of private healthcare sectors resulted in the delivery of poor NCD services with higher costs that ultimately left the poor and disadvantaged groups unable to access the necessary NCD care (29, 55).

In parallel to the above-mentioned challenges, the health system at the PHC level has potential for better managing the NCDs. In line with the global commitment and recommendations, the government of Bangladesh has implemented various policies and strategies, including the NCD-related treatment guidelines and protocols and program implementation plans, devising an essential drug list, multisectoral action plan for NCD prevention, strategic planning for surveillance and prevention, and national communication strategies and action plan (56–59). Translating these guidelines and actions into practice might improve the coverage of NCD services. The health system organization might have the potential for facilitating NCD services at the population level, as it has extensive healthcare infrastructure and is equipped with community health workers to facilitate the preventive and promotive services (12). In recent years, the demand for NCD care increased at the population level, which is an opportunity, as it is an important first step for mobilizing the healthcare services. Capacity development of the large body of informal healthcare providers might be an opportunity for the service delivery (30).

## Strengths and limitations

This study included participants from diverse backgrounds, including program planners, healthcare managers, health service providers, and community members, which enabled the optimization of variation in the participants. Data triangulations gathered through various methods and sources enhanced the trustworthiness of the information provided. Furthermore, the use of the health system dynamics framework provided an analytical basis to comprehensively analyze the health system factors that challenged the organization of NCD service delivery at the PHC level. However, our study has certain limitations that should be acknowledged. A possible limitation is that some participants might have felt uncomfortable about sharing their opinions and thoughts in the group discussions due to the dominant participants. However, the experienced and trained facilitators attempted to minimize this limitation by maintaining good group dynamics during the discussions. The findings of this study are based on data from rural settings; therefore, they may not be easily generalizable across populations and places. Nevertheless, information collected from a diverse group of participants was sufficient to provide an in-depth understanding of factors influencing the preference and willingness of patients to receive services from PHC facilities in preventing and managing NCDs.

## Conclusion

The current study provided insights into the health system challenges in organizing NCD services delivery at the PHC level in Bangladesh. Our findings revealed that although the mobilization of NCD services has been increased at the local healthcare level, a range of health system factors remain a challenge to organize NCD services. Non-adherence to the relevant NCD treatment guidelines and protocols, the dominant presence of under-regulated informal and profit-based private healthcare sectors, poor use of health information and recording, and poor local-level stewardships and coordination systems across healthcare providers and delivery platforms were identified as structural barriers to organizing NCD services. Lack of functional referral services, shortage of medicine supply, limited diagnostic facilities and logistics support, and a large number of untrained healthcare staff weakened the health system at the PHC level. Due to these structural obstacles, the quality of NCD service delivery is being affected. The availability of NCD-related policy documents, the vast network of healthcare infrastructure and frontline staff, and increased demand for NCD services were identified as the major opportunities. These weaknesses need to be addressed to organize the quality NCD services to better respond to the rising burden of NCDs at the PHC level.

## Data availability statement

The data used and analyzed during this research are not publicly available due to ethical restrictions, and data confidentiality. Data are available upon reasonable request from researchers who meet the criteria for access to confidential data. Interested parties may contact AK, md.kabir@monash.edu for further inquiries in this regard.

## Ethics statement

The studies involving human participants were reviewed and approved by Monash University Human Research Ethics Committee (Project ID: 27112) and the Bangladesh Medical Research Council (BMRC) (Ref: BMRC/NREC/2019-2022/270). The patients/participants provided their written informed consent to participate in this study.

## Author contributions

AK and BB conceived and designed the study. AK developed the data collection tools, data collection activities, coordinated the field operations, and prepared the first draft of the manuscript. MK and BB revised the manuscript. BB provided overall stewardship. All authors have read and approved the final manuscript.

## Conflict of interest

The authors declare that the research was conducted in the absence of any commercial or financial relationships that could be construed as a potential conflict of interest.

## Publisher's note

All claims expressed in this article are solely those of the authors and do not necessarily represent those of their affiliated organizations, or those of the publisher, the editors and the reviewers. Any product that may be evaluated in this article, or claim that may be made by its manufacturer, is not guaranteed or endorsed by the publisher.
