# Supplementary material for: Health system challenges and opportunities in organizing non-communicable diseases services delivery at primary healthcare level in Bangladesh: A qualitative study
Source: Front Public Health. 2022 Nov 9;10:1015245. doi: 10.3389/fpubh.2022.1015245 (PMC9682236; doi:10.3389/fpubh.2022.1015245)
Supplement: Supplementary file 1 [file Data_Sheet_1.PDF]

A1: Guidelines for Interviews

Intro questions/background information

I. Background information of the participants (i.e., age, occupation, education, position held, length of service). Would you please tell us about your practice/experience/roles in this facility/area/community? What type of services/care do you provide (i.e., prevention, screening for major NCDs, treatment, rehabilitation/ palliative) (for healthcare providers)?

Healthcare service delivery

II. Would you please tell us about the current status of the NCDs in your catchment areas/ sub-district/districts (i.e., major NCDs, prevalence, most at-risk groups, risk factors, health-seeking process/behaviors of patients with NCDs etc.) (Please discuss elaborately when, how, why, and why not?)

III. Would you please tell us about the current status of the NCDs prevention and control program and service availability (service providers, prevention, treatment, follow-ups, compliance/adherence to service, and rehabilitation) at your facility/catchment areas/subdistrict/districts? When an NCD patient comes, what procedure she/he go through? Who cares for? What type of care?

IV. Would you please tell us about NCDs treatment guidelines and protocols? How often you follow any standard treatment guidelines? (Please discuss elaborately when, how, why, and why not?)

V. Do you receive patients through referrals? Or do you refer patients to others? Can you describe how this referral process work?

Infrastructure and supply

VI. Would you please tell me about infrastructure/facilities, supplies and logistics, diagnostic and equipment available at your clinic/hospital for NCD patients? How do you use these facilities? (Please discuss elaborately when, how, why, and why not?)

VII. How extent do you dispense/prescribe medicines at your clinic/hospital to NCD patients? How extent these prescribed medicines are available at your facility? Does the patients' buying capacity affects NCDs outcomes/health seeking/adherence to treatment (Please discuss elaborately when, how, why, and why not?)

Human resources

VIII. Would you please tell me about the human resource with their qualifications/training and role/responsibility to provide NCD care? (Please discuss elaborately when, how, why, and why not?)

IX. Would you please tell us if the current workforce is adequate for covering NCDs related services to the targeted population (i.e., local-level population group as per sub-districts, district, region) (Please discuss elaborately when, how, why, and why not?)

Availability and use of information system

X. Do you/your facility maintain register exists to monitor follow-up and gather statistics on NCDs (why or why not)?

XI. If yes, how these data help you? (If no data is collected) Do you see any use/need to collect data/information about NCD patients? (why or why not)?

XII. What extent do you use data/scientific evidence available to you when making decisions to guide NCD related policy/prevention, planning, and quality improvement? (i.e., HMIS, performance assessment report, published research studies, national data sources (e.g., morbidity/mortality data), government reports such as BDHS, health survey, facility readiness survey, STEP survey)? (elaborate how, why, why not)?

Leadership, policy, and management

XIII. Do you engage and maintain communication with internal (govt. departments, ministries) and external stakeholders (private hospital, NGOs, informal providers, communities) in managing NCDs? (elaborate how, why, why not)?

XIV. How monitoring, supervision and feedbacks are provided to the respective frontline care providers? (elaborate why, when, why not)?

XV. In your opinion, what strategies/actions are required to strengthen the NCDs related service and care at the primary level care/local level (i.e., from government/public health perspective, NGO/private perspective, community perspective)? (why or why not?)

A2: Guideline for Focused Group Discussion (FGD)

I. Socio-demographic information of the participants (i.e., age, sex, education, occupation, religion, self-reported NCDs, income, type of NCDs developed, length of suffering from the disease/condition)

II. Would you please tell us what the common illnesses are in your area/community? Where people seek care from? Are any difficulties/facilities in seeking care? (Please discuss elaborately when, how, why, and why not?)

III. Do you know anything about NCDs and service availability in the primary healthcare facilitates? (i.e., type of NCDs, reorganization of symptoms, the reason for NCDs, available service at the primary level care facilities anything else.). (Please discuss elaborately when, how, why, and why not?)

IV. Would you please tell us about your NCD service seeking (what type of carers/facility visited, treatment received? (i.e., formal care, informal care, modern medicine/traditional medicine, local/distance, anything else) (Please discuss elaborately when, how, why, and why not?)

V. Do people face any constraints during NCDs' care-seeking process? (i.e., delay in seeking care, discontinuation of care, irregular follow-up/nonadherence, lack of proper information, anything else) (Please discuss elaborately when, how, why, and why not?)

VI. What challenges people face in maintaining continuity of NCD care (current treatment regime, lifestyle modification, coping up with financial hardship, transports/communication) (Please discuss elaborately when, how, why, and why not?)

VII. Would you please tell me about any barriers for NCD care (service fee, out-of-pocket expenditure, medicine cost, availability of supports (i.e., test, pathology), informal fee, user fee, anything else.) (Please discuss elaborately when, how, why, and why not?)

VIII. Please tell us about the enablers of the current NCDs service and care? (i.e., awareness, motivation, adherence to treatment, anything else) (Please discuss elaborately when, how, why, and why not?)

IX. Do people ever felt having benefited from undergone the treatment/screening for NCDs? (Please discuss elaborately when, how, why, and why not)?

X. Would you please tell us about the actions required to strengthen the NCDs related service and care at the primary care settings/local level (i.e., from government/public health perspective, NGO/private perspective, community perspective)? (why or why not?
