# Supplementary material for: Health system challenges and opportunities in organizing non-communicable diseases services delivery at primary healthcare level in Bangladesh: A qualitative study
Source: Front Public Health. 2022 Nov 9;10:1015245. doi: 10.3389/fpubh.2022.1015245 (PMC9682236; doi:10.3389/fpubh.2022.1015245)
Supplement: Supplementary file 2 [file Data_Sheet_2.PDF]

COREQ (Consolidated criteria for Reporting Qualitative research) Checklist

Topic	Item No.	Guide Questions/Description	Remark	Reported on Page No.
Domain 1: Research team and reflexivity				
Personal characteristics				
Interviewer/facilitator	1	Which author/s conducted the interview or focus group?	Lead author, research assistants	Methods – 6-7
Credentials	2	What were the researcher's credentials? E.g. PhD, MD	As mentioned on the title page	Title page - 1
Occupation	3	What was their occupation at the time of the study?	Lead Author: PhD student	Title page - 1
Gender	4	Was the researcher male or female?	Female: 2; Male:2	N/A
Experience and training	5	What experience or training did the researcher have?	The lead author and other co-authors are experienced in conducting both qualitative research designs.	N/A
Relationship with participants				
Relationship established	6	Was a relationship established prior to study commencement?	No relationship with the participants was established before the commencement of the study.	N/A
Participant knowledge of the interviewer	7	What did the participants know about the researcher? e.g. personal goals, reasons for doing the research	The data collection team introduced themselves to participants stating they are public health practitioners and researchers interested to know the health system challenges in organizing non-communicable disease services from primary healthcare facilities. Additionally, they described the purpose of the project and answered any questions participants may have had about the study and those involved in it.	Method-5-6
Interviewer characteristics	8	What characteristics were reported about the inter viewer/facilitator? e.g. Bias, assumptions, reasons and interests in the research topic	The interviewers were public health practitioners with master's degrees in social science, public health, and medicine. They have extensive experience in conducting qualitative research in Bangladesh in the context of health-seeking benthivores, health system factors, and primary healthcare, etc., They had also had extensively read the	Title Page-2

			literature on health system research and service delivery for noncommunicable diseases.	
Domain 2: Study design				
Theoretical framework				
Methodological orientation and Theory	9	What methodological orientation was stated to underpin the study? e.g. grounded theory, discourse analysis, ethnography, phenomenology, content analysis	Inductive thematic analysis	Methods – 7-8
Participant selection				
Sampling	10	How were participants selected? e.g. purposive, convenience, consecutive, snowball	Purposive selection	Methods – 6-7
Method of approach	11	How were participants approached? e.g. face-to-face, telephone, mail, email	Face-to-face	Methods – 6-7
Sample size	12	How many participants were in the study?	Total Number of participants= 106 (92 participants in 16 FGDs, and 14 KIIs participants	Result – 9-19 Table -1, Table - 2
Non-participation	13	How many people refused to participate or dropped out? Reasons?	None	N/A
Setting				
Setting of data collection	14	Where was the data collected? e.g. home, clinic, workplace	Home/workplace	Methods 6-7
Presence of nonparticipants	15	Was anyone else present besides the participants and researchers?	We asked participants to be in a quiet location where they would not be disturbed. However, in some cases, we could not prevent a family member from occasionally disturbing the interview.	N/A
Description of sample	16	What are the important characteristics of the sample? e.g. demographic data, date	As reported in the result section	Result – 9-19 Table -1, Table - 2
Data collection				
Interview guide	17	Were questions, prompts, guides provided by the authors? Was it pilot tested?	The interview guides were piloted in a similar setting (outside the original field sites)	Methods – 7
Repeat interviews	18	Were repeat interviews carried out? If yes, how many?	There was no repeat interview.	N/A
Audio/visual recording	19	Did the research use audio or visual recording to collect the data?	All the interviews and FGDs were audio-recorded	Methods – 7

Field notes	20	Were field notes made during and/or after the interview or focus group?	The research assistant took notes during the interview and FGDs	Methods – 7
Duration	21	What was the duration of the interviews or focus group?	The interviews (KIIs) lasted for 30-45 minutes and FGDs lasted for 45-60 minutes on average	Methods – 6-7
Data saturation	22	Was data saturation discussed?	Data saturation was reached when no new data emerged from the interviews.	Methods – 6
Transcripts returned	23	Were transcripts returned to participants for comment and/or correction?	No	N/A
Domain 3: analysis and findings				
Data analysis				
Number of data coders	24	How many data coders coded the data?	Three	Methods - 6-7
Description of the coding tree	25	Did authors provide a description of the coding tree?	Yes	Figure: 1
Derivation of themes	26	Were themes identified in advance or derived from the data?	Derived from the data	Results Figure: 1
Software	27	What software, if applicable, was used to manage the data?	No	Methods –6-7
Participant checking	28	Did participants provide feedback on the findings?	No	N/A
Reporting				
Quotations presented	29	Were participant quotations presented to illustrate the themes/findings?<br>Was each quotation identified? e.g. participant number	Yes	Results – 9-19
Data and findings consistent	30	Was there consistency between the data presented and the findings?	Yes	N/A
Clarity of major themes	31	Were major themes clearly presented in the findings?	Yes	Results – 9-19 Figure 1
Clarity of minor themes	32	Is there a description of diverse cases or discussion of minor themes?	Yes	Results – 9-19 Figure 1
